# An Ex Vivo ‘*Leaky Skin*’ Model to Study Early Events Induced by *Staphylococcus aureus* Protease

**DOI:** 10.3390/microorganisms14061244

**Published:** 2026-06-01

**Authors:** Andrea Cavagnino, Olivier Gouin, Lionel Breton, Martin Baraibar

**Affiliations:** 1 OxiProteomics, 2 Rue Antoine Étex, 94000 Créteil, France; 2 CILIA Consulting, 20 Av. De Paris, 78000 Versailles, France

**Keywords:** *Staphylococcus aureus*, SspA, serine protease, ex vivo skin model, epidermal barrier disruption, tight junctions, proteostasis, protein carbonylation, skin dysbiosis, atopic dermatitis

## Abstract

Maintaining a balanced skin microbiota is essential for preserving epidermal barrier integrity and overall skin health. Dysbiosis, particularly the opportunistic overgrowth of *Staphylococcus aureus*, is associated with barrier dysfunction and inflammatory dermatoses such as atopic dermatitis, psoriasis, and acne. In dysbiotic states, microbial regulatory mechanisms become disrupted, enabling pathogenic strains to proliferate and release proteases that degrade structural components of the skin barrier, increasing epidermal permeability in a manner analogous to ‘leaky gut’ physiopathology. Microbiota dysbiosis has further been proposed as an emerging hallmark of aging, contributing to chronic low-grade inflammation, impaired tissue repair, and progressive barrier decline. Current strategies predominantly target the microbiota itself, leaving the host tissue response to protease-mediated barrier disruption comparatively underaddressed. To fill this gap, an ex vivo human skin model was developed based on topical application of purified *S. aureus* serine protease SspA to skin explants, enabling controlled investigation of early host–microbiota interaction events. Barrier function, junctional integrity, inflammatory mediators, and proteostasis were assessed through a panel of complementary biomarkers—Lucifer Yellow permeability, claudin-1, desmoglein-1, filaggrin, IL-31, S100A8/A9, PGE2, and protein carbonylation. SspA induced measurable barrier disruption, junctional protein loss, inflammatory mediator upregulation, and proteostasis impairment without overt tissue damage. A biotic culture filtrate of *Bifidobacterium adolescentis* partially attenuated SspA-induced protein carbonylation. This model provides the scientific community with a controlled, biologically relevant platform for identifying biomarkers of early barrier impairment and evaluating host-targeted interventions aimed at preventing or counteracting protease-driven barrier damage in dysbiosis-associated skin conditions. A better understanding of the early molecular mechanisms through which microbial virulence factors drive barrier disruption and proteostasis decline may further contribute to broader strategies aimed at preserving skin integrity during aging.

## 1. Introduction

The skin microbiota and the epidermal barrier function as an integrated system, where homeostatic balance between host tissue and resident microbial communities is critical for maintaining skin health [1,2]. Perturbation of this equilibrium, in particular through opportunistic expansion of *Staphylococcus aureus*, has been consistently linked to epidermal barrier dysfunction and the pathogenesis of inflammatory skin conditions [3]. As the body’s primary interface with the external environment, the skin relies on the structural integrity of the epidermis and the coordinated activity of innate immune defenses and commensal microorganisms to resist pathogenic invasion and environmental insults [4,5]. Barrier integrity may be compromised by a range of extrinsic and intrinsic factors, including UV radiation, temperature fluctuations, psychological stress, nutritional status, and systemic physiological conditions [6,7].

While most skin-resident microorganisms are harmless or commensal [5,8], some—most notably *S. aureus*—can shift toward pathogenicity under permissive conditions. Although asymptomatic carriage occurs in a minority of healthy individuals, its prevalence rises dramatically in inflammatory skin conditions: *S. aureus* is detected on lesional skin in the vast majority of atopic dermatitis patients, and has also been documented in psoriasis and acne vulgaris [9,10,11]. Across these conditions, the burden of colonization correlates with disease severity and clinical exacerbation [10]. The skin microbiota operates as a dynamic ecological system in which processes such as biofilm formation and quorum sensing play key roles in maintaining microbial balance and host compatibility [12,13]. Under dysbiotic conditions, however, these regulatory mechanisms may fail, enabling pathogenic strains to proliferate, outcompete commensal populations, and drive tissue degradation through upregulated protease activity [14,15]. The functional consequences of such epidermal barrier failure share conceptual parallels with intestinal hyperpermeability, the so-called ‘leaky gut’, in that structural compromise of the epithelial layer allows passage of microorganisms, pro-inflammatory molecules, and sensitizing agents that would otherwise be excluded [16,17]. Through systemic circulation, such translocation events may propagate inflammatory signals beyond the skin, with potential consequences for distant tissues and organ systems [16,17]. *S. aureus* colonization is a prominent feature of several inflammatory skin disorders characterized by epidermal barrier dysfunction, most notably atopic dermatitis (AD), but also psoriasis and xerotic skin conditions. Increasing evidence indicates that *S. aureus* contributes directly to disease pathogenesis through the secretion of virulence factors capable of altering epidermal integrity and modulating host immune responses [3,9]. Among these, secreted serine proteases have emerged as major effectors of host–pathogen interaction and barrier damage [14,15].

The *S. aureus* extracellular protease system includes the serine protease SspA (also known as V8 protease), which plays a central role in the proteolytic cascade by activating downstream proteases and contributing to tissue damage and immune modulation [14,18,19]. SspA can degrade host proteins involved in epidermal cohesion and barrier maintenance, thereby facilitating bacterial persistence and amplifying inflammatory signaling within the skin microenvironment [14,18,20]. Exposure of keratinocytes to SspA and related *S. aureus* products triggers the release of multiple inflammatory mediators, including cytokines and alarmins such as IL-1β, IL-6, and IL-8 [21,22], as well as damage-associated molecules S100A8 and S100A9 [23,24]. These mediators promote leukocyte recruitment and amplify innate immune signaling through pattern recognition receptors such as TLR4 and RAGE [23,24]. In parallel, keratinocyte stimulation can induce cyclooxygenase-2 (COX-2) expression and prostaglandin E_2_ (PGE_2_) production, further contributing to inflammatory responses and immune modulation in the epidermis [21]. Furthermore, *S. aureus*–mediated epithelial stress has been associated with upregulation of IL-31, a pruritogenic cytokine involved in the itch–scratch cycle that contributes to disease chronicity by exacerbating barrier damage and inflammation [25]. Collectively, these observations indicate that SspA acts not only as a tissue-degrading enzyme but also as an important modulator of host epithelial signaling, capable of initiating and sustaining a self-amplifying inflammatory program. In this study, we focused on SspA, which is significantly upregulated during atopic dermatitis and may also be relevant in other barrier-compromised conditions, including psoriasis and xerosis. In vivo murine studies have shown that SspA compromises skin integrity by damaging the stratum corneum, thereby weakening the primary physical barrier [26,27]. Beyond effects on the outermost layers, SspA has also been associated with tight junction disruption and global impairment of barrier function, mechanisms thought to result from keratinocyte and epithelial stress [27,28,29,30]. Collectively, these observations support the use of SspA as a biologically relevant and mechanistically controlled trigger for reproducing early protease-mediated barrier impairment and capturing the initial molecular events preceding sustained inflammation.

At the molecular level, disruption of skin homeostasis during dysbiosis is reflected by alterations in key structural and functional biomarkers of the epidermal barrier. Among the structural determinants of epidermal barrier integrity, claudin-1 is a key tight junction component that governs paracellular sealing within the stratum granulosum; its loss has been associated with increased permeability and heightened susceptibility to *S. aureus* colonization [29,31]. Desmoglein-1, a desmosomal cadherin essential for corneocyte adhesion in the upper epidermis, represents another critical target of *S. aureus* proteases, whose degradation compromises mechanical cohesion and facilitates microbial access to deeper tissue layers [27,32]. Filaggrin deficiency, widely recognized as a primary risk factor for atopic dermatitis, impairs stratum corneum maturation, reduces natural moisturizing factor levels, and creates a microenvironment that favors *S. aureus* colonization and dysbiosis [33,34]. Together, these three proteins constitute interdependent structural nodes whose simultaneous assessment provides a sensitive and integrated readout of early barrier deterioration. Beyond structural markers, early barrier impairment can be assessed through functional and biochemical readouts. Paracellular permeability was evaluated using Lucifer Yellow, a hydrophilic membrane-impermeant fluorescent tracer whose diffusion across the epidermal layers is restricted under conditions of intact barrier function, increased penetration therefore reflecting intercellular junctional impairment. In parallel, protein carbonylation, reflecting stable ROS-mediated modifications of amino acid side chains, was used as a tissue-level integrative indicator of oxidative burden and proteostasis impairment [35,36,37]. These complementary readouts provide an integrated view of barrier function, epithelial integrity, immune activation, redox status, and proteostasis, enabling sensitive detection of early skin damage induced by SspA.

Current strategies targeting microbiota-associated skin conditions predominantly focus on modulating microbial populations directly, while the host tissue response to microbial virulence factors, particularly protease-mediated barrier disruption and its downstream molecular consequences, remains comparatively underaddressed. Conditions such as skin aging, atopic or senile xerosis, and sensitive or dry skin are frequently associated with microbiota imbalance and manifest as increased permeability, chronic low-grade inflammation, and heightened sensitivity to external aggressors [1,2,38]. A model targeting the host-side molecular events, rather than the microbiota itself, may therefore offer a complementary platform for identifying interventions capable of preserving or restoring barrier integrity in dysbiosis-associated skin conditions. Chronic inflammation (“inflammaging”), loss of proteostasis, and microbiota dysbiosis are increasingly recognized as interconnected hallmarks of aging that contribute to progressive tissue dysfunction [37,38,39]. A better understanding of the early molecular mechanisms through which microbial virulence factors drive barrier disruption and proteostasis decline may therefore contribute to broader strategies aimed at preserving skin integrity during aging.

Despite growing interest in microbiota–skin interactions, experimental models for studying *S. aureus*–mediated barrier disruption remain limited. In vitro colonization models often exhibit high variability due to differences in bacterial growth dynamics and host tissue heterogeneity and are frequently incompatible with the testing of formulated products. Reconstructed human epidermis (RHE) models offer improved standardization but lack the full tissue architecture and immune context of native skin. In vivo models, while physiologically relevant, involve longer timelines and raise ethical and regulatory constraints, limiting their utility for early-stage mechanistic screening. In this context, ex vivo human skin explants provide a controlled and biologically relevant alternative, enabling the study of host–pathogen interactions in preserved native tissue while maintaining experimental reproducibility [40]. The use of a defined virulence factor such as SspA, rather than live bacteria, further enhances mechanistic control and reproducibility, allowing dissection of the specific molecular events attributable to protease activity.

To advance our understanding of early protease-mediated barrier disruption, an ex vivo human skin model was developed based on topical application of purified SspA to skin explants. Using a panel of complementary biomarkers, including Lucifer Yellow permeability, claudin-1, desmoglein-1, filaggrin, IL-31, S100A8/A9, PGE2, and protein carbonylation, the earliest molecular events induced by SspA activity were characterized and relevant biomarkers of barrier impairment identified. Beyond its applicability as a proof-of-concept screening platform for protective or repairing interventions, this model may also contribute to the emerging field of longevity science, as proteostasis disruption and microbiota-driven barrier dysfunction are increasingly recognized as interconnected drivers of skin aging and tissue resilience decline [37,38,39].

## 2. Materials and Methods

### 2.1. Ex Vivo 3D Models of Skin

Human skin explants were obtained from abdominal surgery of a single female Caucasian donor, with written informed consent obtained prior to tissue collection. Explants were distributed into two experimental groups (*n* = 6 per group) and maintained under standard culture conditions in a CO_2_-humidified incubator. Following an initial equilibration period, explants were topically treated (30 µL/cm^2^) once per day for two consecutive days with either: (i) purified SspA serine protease (0.01 µg/mL; Cusabio Technology LLC, Houston, TX, USA); or (ii) dilution buffer alone (negative control). Culture medium was renewed every 24 h. For the assessment of the *B. adolescentis* culture filtrate, reconstructed human epidermis models (EpiSkin™, Lyon, France) were used. Three experimental conditions were applied (*n* = 3 per condition): (i) dilution buffer alone (negative control); (ii) SspA (0.01 µg/mL); or (iii) SspA (0.01 µg/mL) combined with *B. adolescentis* culture filtrate (10% *v*/*v*). Treatment conditions and duration were identical to those applied to skin explants. Protein carbonylation was assessed as a primary readout for this experiment.

Culture medium was renewed every 24 h. The SspA concentration was intentionally set well below the 3 µg/mL threshold reported to induce significant barrier damage in keratinocyte models [30], to capture subtle early molecular events preceding overt tissue disruption. The *B. adolescentis* culture filtrate consisted of the culture medium recovered following standard cultivation under typical fermentation conditions, gratuitously provided by the originating laboratory (Telostim, Paris, France) and stored at −20 °C until use. The 10% (*v*/*v*) concentration was selected on the basis of prior topical application studies on human skin conducted by the originating laboratory.

At the end of the treatment period, explants were allocated to two analytical workflows. Three explants per group received topical application of Lucifer Yellow solution (1 mg/mL; L0144, Sigma-Aldrich-Merck KGaA, Darmstadt, Germany), incubated for one hour to assess paracellular permeability, then embedded in OCT compound (Cryomatrix™, Thermo Fisher Scientific, Asnières-sur-Seine, France), snap-frozen in liquid nitrogen, and stored at −80 °C until cryosectioning. The remaining three explants per group were each bisected: one half was embedded in OCT compound, snap-frozen in liquid nitrogen, and stored for cryosectioning and histological analyses; the other half was snap-frozen in liquid nitrogen and stored at −80 °C for biochemical analyses.

### 2.2. Detection, Visualization, and Quantification of Biomarkers on Skin Sections

Cryosections of 5 µm thickness were obtained from snap-frozen skin explants using a cryostat (Leica, Wetzlar, Germany) and fixed using a standard fixation protocol as previously reported [41,42]. Sections from Lucifer Yellow-treated explants were analyzed for epidermal barrier integrity by measuring the specific fluorescent signal intensity (Ex. 428 nm/Em. 536 nm) at 250 µm from the explant surface. In situ protein carbonylation was detected as previously described [35,36,41,42].

For immunofluorescence detection, non-specific sites were blocked with bovine serum albumin (BSA; Sigma-Aldrich-Merck) in PBS. Tissue sections were subsequently incubated with primary antibodies directed against each target biomarker, followed by incubation with the appropriate fluorophore-conjugated secondary antibody (Table 1). Cell nuclei were counterstained with DAPI (4′,6-diamidino-2-phenylindole). Washing steps after antibody incubations were performed with PBS/BSA solutions. Fluorescent images were acquired using an epifluorescence microscope (EVOS M5000 or M7000 Imaging System, Thermo Fisher Scientific, Asnières-sur-Seine, France) and quantified using ImageJ software (version 1.53, National Institutes of Health, Bethesda, MD, USA).

Biomarkers were quantified by image analyses integrating the specific fluorescent signal intensity over the analyzed surface.

To assess tissue morphology and viability across all experimental conditions, H&E, hematoxylin (MHS-16, Sigma-Aldrich-Merck KGaA) and eosin (10479814, Thermo Fisher Scientific), staining was performed on cryosections from each experimental group. Representative images are provided as Supplementary Figure S1.

### 2.3. ELISA Assays

The half-explants allocated to biochemical analyses were subjected to protein extraction using the optimized OxiProteomics^®^ buffer (OxiProteomics, Créteil, France). Total protein concentration was determined using the Bradford Protein Assay Dye Reagent (Bio-Rad™, Marnes-la-Coquette, France) according to the manufacturer’s guidelines. PGE2 levels were measured using a commercial ELISA kit (EHPGE2, Invitrogen, Thermo Fisher Scientific, Asnières-sur-Seine, France) according to the manufacturer’s guidelines. Absorbance was measured using a Varioskan plate reader (Thermo Fisher Scientific, Asnières-sur-Seine, France). PGE2 concentrations were normalized to total protein content.

### 2.4. Data Integration and Statistics

For each experimental condition, one cryosection was obtained from each of the three independent skin explants per condition allocated to immunofluorescence or permeability analyses, or three independent RHE per condition, yielding three independent measurements per condition (*n* = 3). Where multiple microscopy fields were acquired from a single section, values were averaged to generate one representative value per explant prior to statistical analysis. For biochemical analyses, one PGE2 measurement was performed per protein extract, prepared independently from the snap-frozen half of each of the three remaining explants, yielding *n* = 3 per condition. Biomarker quantifications were normalized relative to the buffer control condition, set at 100%, and results are expressed as mean ± standard deviation.

Statistical analyses were performed using GraphPad Prism software (version 10.0.3, GraphPad Software, La Jolla, CA, USA). For pairwise comparisons, unpaired *t*-test was applied. For multiple group comparisons, one-way ANOVA followed by Dunnett’s post hoc test was used, with the SspA-treated (‘leaky skin’) group as the reference condition. Statistical significance thresholds were defined as follows: * *p* < 0.05, ** *p* < 0.01, *** *p* < 0.001; ns, not significant.

## 3. Results

### 3.1. Functional Impairment of Skin Barrier

**Figure 1:**
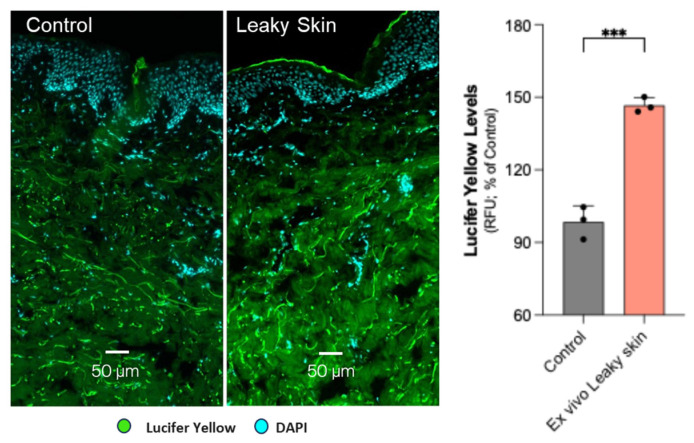
Increased penetration of a fluorescent dye (visualized in green) upon SspA treatment (labelled Leaky Skin in the images), indicating functional impairment of skin barrier integrity and permeability. The quantification of fluorophore penetration is measured as fluorescence intensity (RFU) at 250 µm from the skin surface and shown as graph bars representing mean ± SD, normalized versus the control. Statistics: (*n* = 3, individual data points are displayed as dot plots; unpaired *t*-test; *** *p* < 0.001). Scale bar 50 µm. Images were acquired with a 10× objective.

Topical application of SspA significantly compromised skin barrier integrity, as evidenced by increased Lucifer Yellow paracellular permeability compared with the buffer control (Figure 1).

**Figure 2:**
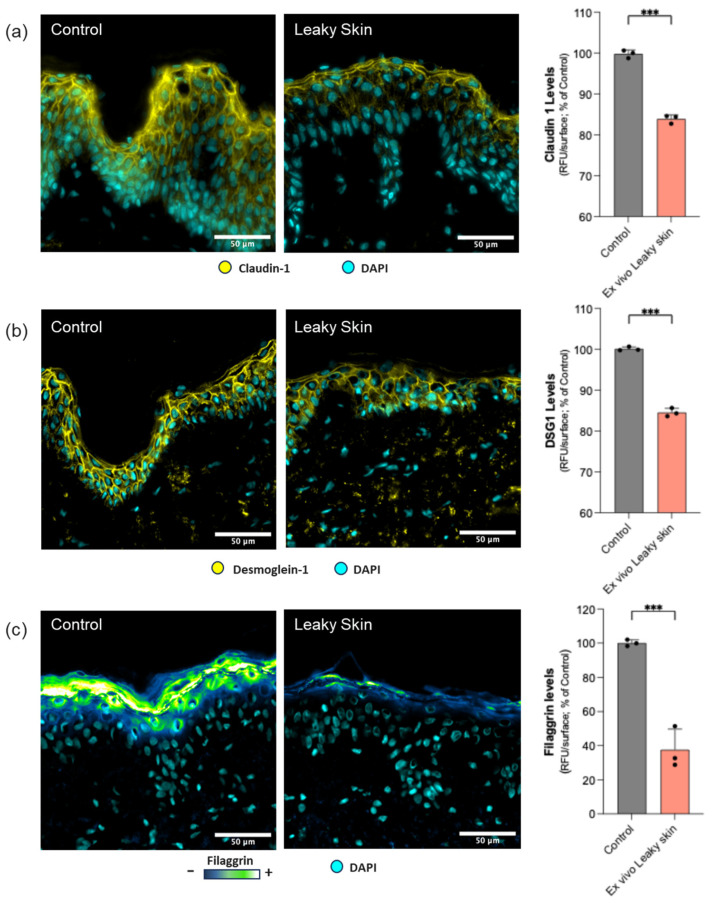
In situ (on skin explant sections) visualization of claudin-1 (yellow, (**a**)), desmoglein-1 (yellow, (**b**)), filaggrin (color range, (**c**)), upon SspA exposure (labelled Leaky Skin in the images). Nuclear detection is shown in cyan (DAPI). The quantification of each biomarker (relative fluorescence intensity over the surface) is shown as bar graphs representing mean ± SD, normalized versus the control condition. Statistics: (*n* = 3, individual data points are displayed as dot plots; unpaired *t*-test; *** *p* < 0.001). Scale bar 50 µm. Images were acquired with a 40× objective.

Consistent with functional barrier impairment, significant reductions in claudin-1 (Figure 2a), desmoglein-1 (Figure 2b), and filaggrin (Figure 2c) levels were observed following SspA exposure, providing a molecular signature of protease-induced epidermal barrier damage across tight junction, desmosomal, and cornified envelope compartments.

### 3.2. Inflammatory Response

**Figure 3:**
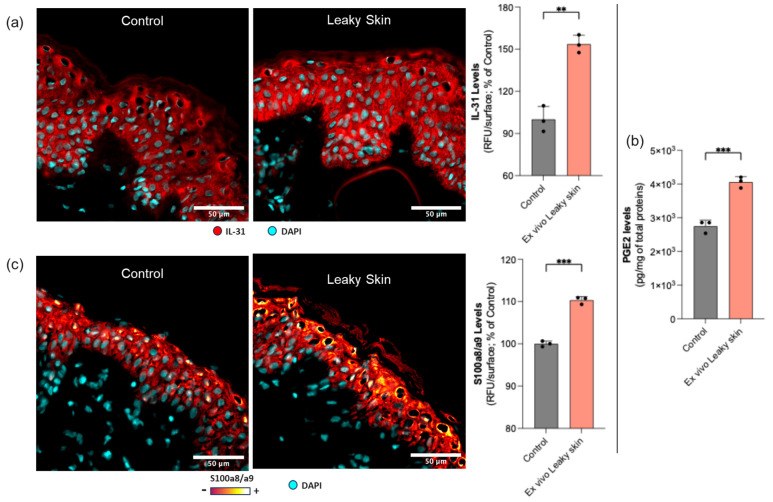
In situ (on skin explant sections) visualization of IL-31 (in red; nuclear detection in cyan, DAPI; (**a**) PGE_2_ levels, (**b**) and S100A8/A9 levels (in color range, red lower levels, bright color high levels; nuclear detection in cyan; (**c**) upon SspA exposure (labelled Leaky Skin in the images). The quantification of Il-31 and S100A8/A9 (as relative fluorescence intensity over the surface) or PGE2 (ng/mg of total proteins) is shown as graph bars representing mean ± SD. Statistics: (*n* = 3, individual data points are displayed as dot plots; unpaired *t*-test; *** *p* < 0.001, ** *p* < 0.01). Scale bar 50 µm. Images were acquired with a 40× objective.

SspA exposure induced a significant upregulation of IL-31 (Figure 3a), PGE2 (Figure 3b), and S100A8/A9 (Figure 3c) compared with the buffer control, indicating activation of early inflammatory and innate immune pathways in the treated tissue.

### 3.3. Oxidative Stress and Proteostasis Impairment

**Figure 4:**
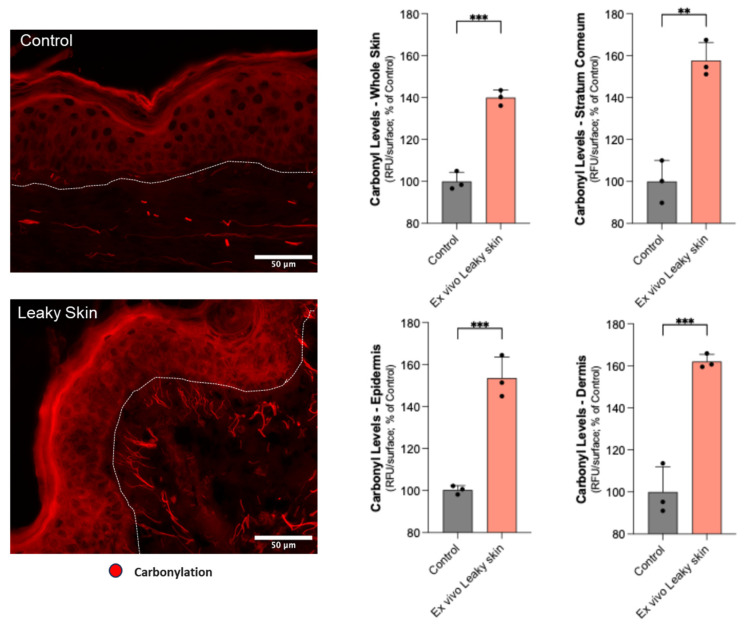
In situ (on skin explant sections) visualization of carbonylation levels (red), upon SspA exposure (labelled Leaky Skin in the images). Carbonylation levels were evaluated in the overall skin section or by individual anatomical compartment (stratum corneum, epidermis, dermis), indicating the deeper impact of SspA-induced barrier dysfunction. Epidermis and dermis are separated by a white dotted line. The quantification of carbonylation levels (as relative fluorescence intensity over the surface) is shown as graph bars representing mean ± SD. Statistics: (*n* = 3, individual data points are displayed as dot plots; unpaired *t*-test; *** *p* < 0.001, ** *p* < 0.01). Scale bar 50 µm. Images were acquired with a 40× objective.

Following SspA exposure, direct fluorescent labeling of carbonylated proteins increased significantly compared with the buffer control (Figure 4). Protein carbonylation, reflecting irreversible ROS-mediated modifications of amino acid side chains, was used here as a tissue-level integrative indicator of oxidative burden and proteostasis impairment [35,36]. Quantitative image analysis confirmed this increase both at the whole skin section level and when stratifying by anatomical compartment (stratum corneum, epidermis, dermis), indicating that the oxidative response was not restricted to a single layer but extended across barrier and deeper tissue regions.

### 3.4. Effect of B. adolescentis Culture Filtrate on Protein Carbonylation

**Figure 5:**
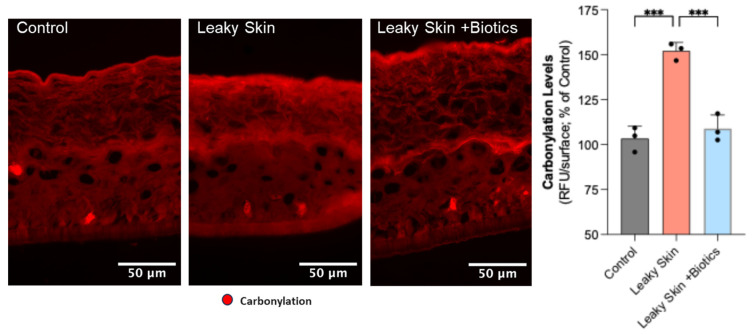
In situ (on RHE sections) visualization of carbonylation levels upon treatments (in red). The quantification of carbonylation levels on stratum corneum layers (as relative fluorescence intensity over the surface) is shown as graph bars representing mean ± SD. Statistics (*n* = 3, individual data points are displayed as dot plots; ANOVA and Dunnett’s post hoc test versus “Leaky Skin” group; *** *p* < 0.001). Scale bar 50 µm. Images were acquired with a 40× objective.

Co-treatment of RHE with SspA and *B. adolescentis* culture filtrate (“Biotics”; 10% *v*/*v*) markedly reduced protease-induced protein carbonylation compared with SspA treatment alone (Leaky Skin, Figure 5), indicating preserved protein homeostasis at the tissue level.

## 4. Discussion

Topical application of SspA to human skin explants induced a convergent pattern of epidermal barrier alterations, affecting functional permeability, structural junctional proteins, inflammatory mediators, and proteostasis simultaneously. The concurrence of these changes across distinct biological readouts suggests that SspA does not act through a single mechanism but rather engages multiple interdependent components of barrier homeostasis within a short, controlled exposure window.

The significant reduction in claudin-1, desmoglein-1, and filaggrin confirms that SspA targets tight junctions, desmosomes, and the cornified envelope in parallel. This is consistent with the “leaky barrier” concept described in epithelial biology, whereby junctional failure and impaired keratinocyte differentiation create permissive conditions for microbial penetration and sustained inflammatory activation. The concurrent loss of these three proteins, rather than individually, reflects the interdependence of epidermal structural compartments and suggests that even low-dose protease exposure is sufficient to unsettle barrier architecture before overt tissue damage occurs, as confirmed by histological assessment (Supplementary Figure S1).

The induction of IL-31, S100A8/A9, and PGE2 indicates that structural damage and inflammatory signaling occur in parallel rather than sequentially. IL-31 upregulation is notable given its established association with pruritus and type 2-skewed inflammation in atopic dermatitis, suggesting that the model captures a cytokine signature relevant to early disease-associated events. The rise in S100A8/A9 reflects keratinocyte danger signaling, while elevated PGE2, a downstream product of arachidonic acid metabolism, points to activation of lipid mediator pathways known to amplify local inflammatory responses and sensitization. Taken together, these three mediators indicate that SspA-induced barrier injury extends beyond structural disruption into early innate immune activation.

Protein carbonylation increased significantly following SspA exposure, distributed across all three skin compartments: stratum corneum, epidermis, and dermis. This distribution suggests that the oxidative response is not confined to the outermost barrier layers but propagates into deeper tissue, with potential implications for the integrity of dermal components. Protein carbonylation reflects irreversible oxidative modification of amino acid side chains and is recognized as a marker of proteostasis impairment and a hallmark of cellular aging [37,38,39]. The detection of elevated carbonylation levels after acute SspA exposure, in the absence of overt cytotoxicity, points to proteostasis disruption as an early and sensitive consequence of protease-mediated barrier insult, a link not previously demonstrated in human skin tissue to our knowledge.

RHE systems have been used in dysbiosis-related research and *S. aureus* colonization assays and evaluation of skin care ingredients in inflammatory skin contexts [43,44], providing an initial methodologically recognized framework within which the protective effect of the *B. adolescentis* culture filtrate was assessed. Consistent induction of protein carbonylation across both skin explants and reconstructed epidermis confirms that the protease-induced oxidative response is reproducible, independent of donor-specific tissue characteristics, reinforcing the biological robustness of the protease challenge. The observed reduction in SspA-induced carbonylation on RHE is consistent with the documented antioxidant properties of *B. adolescentis* culture filtrates [45] and raises the possibility that secreted bacterial metabolites contribute to attenuation of protease-induced oxidative stress [46]. The full characterization of the active fraction(s) responsible for this protective effect, as well as its impact on additional barrier-related biomarkers, remains a perspective for future studies.

The present ex vivo organotypic model presents several features that distinguish it from existing approaches. Human skin explants retain the full tissue architecture, including dermal components, immune cells, and stromal context, that reconstructed epidermis models inherently lack. The use of a defined, purified protease at sub-cytotoxic concentrations provides mechanistic specificity, avoiding the interpretive complexity associated with live bacteria or crude bacterial supernatants. The biomarker panel, spanning functional, structural, inflammatory, and oxidative readouts, enables detection of early, pre-destructive barrier changes that would be missed by single-endpoint assays. These features make the model particularly suited to evaluating host-targeted interventions rather than antimicrobial strategies, addressing a gap in current experimental approaches where most platforms are oriented toward microbiota modulation rather than the host tissue response. Current strategies in this field tend to focus on modulating microbiota composition, through probiotic, prebiotic, or antimicrobial approaches, while the host tissue response to specific microbial virulence factors has received comparatively less attention, opening the field to evaluation of benefits mediated by other classes of compounds or biological interventions. By focusing on a defined protease and measuring its impact on host tissue directly, this model offers a different experimental approach and may be useful for evaluating ingredients or formulations that act on barrier resilience rather than microbial ecology.

The single-donor design invites multi-donor validation to characterize inter-individual variability in protease sensitivity, barrier biology, and inflammatory tone; a step supported in part by the consistency of the carbonylation response observed across both ex vivo skin explants and RHE, providing initial evidence that the protease-induced phenotype is not restricted to a single donor background.

The inclusion of protease inactivation or inhibition controls in future iterations would formally confirm that the observed alterations are attributable to SspA enzymatic activity specifically, strengthening the mechanistic characterization of the model. It should be noted, however, that the relevance of host-targeted interventions identified through this platform does not depend on direct protease inhibition; compounds acting downstream on barrier structural integrity, redox homeostasis, or inflammatory signaling may confer meaningful biological benefit independently of any effect on SspA activity itself. A dose–response characterization would map the biological response curve and identify threshold concentrations for distinct phenotypic outcomes, from subtle early alterations to progressive barrier failure, enabling model calibration for specific research questions. The two-day exposure window, chosen here to capture early molecular events, could be extended or repeated to model subchronic or chronic protease exposure scenarios. Future iterations could also incorporate additional components of the *S. aureus* secretome alongside SspA, providing a more integrated representation of protease-mediated host–pathogen interactions. Finally, the *B. adolescentis* culture filtrate, limited to protein carbonylation as a readout, opens the door to a broader characterization of protective potential across the full biomarker panel, including functional permeability, junctional proteins, and inflammatory mediators, and specifically to the potential identification of its active fraction(s) responsible for the observed antioxidant effect.

The obtained results confirm the initial objectives of this work. Topical application of purified SspA to ex vivo human skin explants induced early, significant alterations across functional, structural, inflammatory, and proteostasis-related readouts. The identification of protein carbonylation as an early tissue-level consequence of SspA activity, alongside established markers of junctional and inflammatory dysregulation, provides a mechanistically grounded set of biomarkers for future interventional studies and supports the biological relevance of this model as a platform for studying host tissue responses to microbial protease activity.

## 5. Conclusions

Topical application of purified SspA to ex vivo human skin explants induced early, measurable alterations across functional, structural, inflammatory, and proteostasis-related readouts, collectively supporting the biological relevance of the model as a platform for studying early protease-mediated barrier disruption, a “leaky skin” model.

The identification of protein carbonylation as an early tissue-level consequence of SspA activity, alongside established markers of junctional and inflammatory dysregulation, provides a mechanistically grounded set of biomarkers for future interventional studies.

This ex vivo “leaky skin” model provides a proof-of-concept platform for: (i) identifying early biomarkers of SspA-induced barrier disruption; (ii) dissecting the contribution of specific virulence factors to epidermal damage; and (iii) evaluating host-targeted protective and/or recovery interventions relevant to dysbiosis-associated skin conditions, with potential for extension as a broader screening tool as validation progresses across multiple donors, exposure conditions, and biomarker endpoints.

These findings are consistent with the emerging recognition of proteostasis disruption and microbiota-driven barrier dysfunction as interconnected early drivers of skin tissue decline [37,38,39], and support the relevance of this model for investigations linking microbial protease activity to skin aging biology, an area warranting further study in the context of longevity science.

## Figures and Tables

**Table 1:** List of antibodies.

Description	Target and Reference
	Claudin-1 (Santa Cruz Biotechnology ^1^, sc-81796)
	Desmoglein-1 (Invitrogen, 32-6000)
Primary antibodies	Filaggrin (Santa Cruz Biotechnology, sc-66192)
	Biotin Labeled IL-31 (BioLegend ^2^, 530004)
	S100A8/A9 (Abcam ^3^, ab22506)
Secondary antibody	Anti-Mouse Alexafluor 647 (Invitrogen A21235)

^1^ Dallas, TX, USA. ^2^ Pietersbergweg, Amsterdam, The Netherlands. ^3^ Cambridge, UK.

## Data Availability

The original contributions presented in this study are included in the article/Supplementary Materials. Further inquiries can be directed to the corresponding authors.

## Supplementary Materials

The following supporting information can be downloaded at: https://www.mdpi.com/article/10.3390/microorganisms14061244/s1, Figure S1: Hematoxylin and eosin (H&E) staining performed on cryosections from buffer control and SspA-treated (“Leaky Skin”) skin explants.
