# Supplementary material for: An Ex Vivo ‘Leaky Skin’ Model to Study Early Events Induced by Staphylococcus aureus Protease
Source: Microorganisms. 2026 Jun 1;14(6):1244. doi: 10.3390/microorganisms14061244 (PMC13304451; doi:10.3390/microorganisms14061244)
Supplement: Supplementary file 1 [file microorganisms-14-01244-s001.zip › microorganisms-4303702-supplementary.pdf]

Supplementary Material

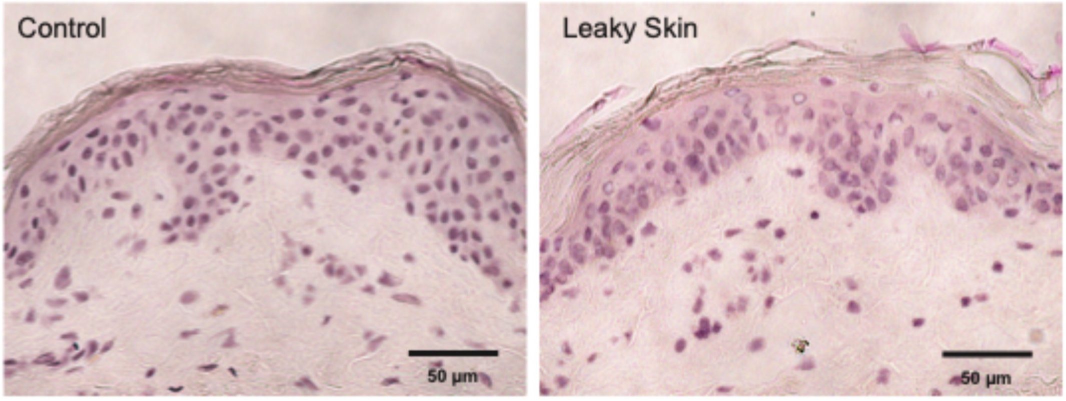

Figure S1. Hematoxylin and eosin (H&E) staining performed on cryosections from buffer control and SspA-treated ("Leaky Skin") skin explants. The absence of visible tissue disorganization, nuclear condensation, or necrotic areas across all conditions confirms that the observed molecular and functional alterations reflect early, controlled barrier disruption rather than nonspecific cytotoxicity. Representative images are shown. Scale bar = 50 µm. Images were acquired with a 40x objective.
